# CDC42 Is Required for Tissue Lamination and Cell Survival in the Mouse Retina

**DOI:** 10.1371/journal.pone.0053806

**Published:** 2013-01-23

**Authors:** Severin Reinhard Heynen, Isabelle Meneau, Christian Caprara, Marijana Samardzija, Cornelia Imsand, Edward M. Levine, Christian Grimm

**Affiliations:** 1 Laboratory of Retinal Cell Biology, Ophthalmology Department, University of Zurich, Switzerland; 2 Zurich Center for Integrative Human Physiology, University of Zurich, Switzerland; 3 Department of Ophthalmology and Visual Sciences, John A. Moran Eye Center, University of Utah, Salt Lake City, Utah, United States of America; 4 Center for Neuroscience, University of Zurich, Switzerland; Center for Regenerative Therapies Dresden, Germany

## Abstract

The small GTPase CDC42 has pleiotropic functions during development and in the adult. These functions include intra- as well as intercellular tasks such as organization of the cytoskeleton and, at least in epithelial cells, formation of adherens junctions. To investigate CDC42 in the neuronal retina, we generated retina-specific *Cdc42*-knockdown mice (*Cdc42-KD*) and analyzed the ensuing consequences for the developing and postnatal retina. Lack of CDC42 affected organization of the developing retina as early as E17.5, prevented correct tissue lamination, and resulted in progressive retinal degeneration and severely reduced retinal function of the postnatal retina. Despite the disorganization of the retina, formation of the primary vascular plexus was not strongly affected. However, both deeper vascular plexi developed abnormally with no clear layering of the vessels. Retinas of *Cdc42-KD* mice showed increased expression of pro-survival, but also of pro-apoptotic and pro-inflammatory genes and exhibited prolonged Müller glia hypertrophy. Thus, functional CDC42 is important for correct tissue organization already during retinal development. Its absence leads to severe destabilization of the postnatal retina with strong degeneration and loss of retinal function.

## Introduction

The sensory retina contains six neuronal cell classes and one glial cell type (Müller glia), which originate from retinal progenitors during retinal development. Although astrocytes and microglia are additional cells important for retinal physiology, they arise from other progenitor populations, as do the cells that make up the retinal vasculature [1]–[3]. All cells are arranged in a highly organized, multi-laminar tissue structure positioning individual cell types at specific, stereotypical locations in the mature retina. Cone and rod photoreceptor cell bodies are found in the outer nuclear layer (ONL), nuclei of horizontal, bipolar, amacrine and Müller glia cells reside in the inner nuclear layer (INL), and ganglion and displaced amacrine cells occupy the ganglion cell layer (GCL). Astrocytes and microglia are found mainly in the vicinity of vessels in the primary plexus and in the plexiform layers, respectively. Correct localization of cells in the mature retina involves movement of retinal progenitor cells through the neuroepithelium during development, interkinetic nuclear migration and cell fate decision processes [4]. Anchoring cell processes by junction and adhesion proteins during these processes is important for accurate development and cell placement. In the adult retina, adherens junctions in the outer limiting membrane (OLM) connect the actin cytoskeleton of photoreceptors and neighboring Müller glia cells, which improves tissue stability and organization of the ONL [5]. In addition, adherens junctions were shown to be important for establishing cell polarity [6], a feature especially important for photoreceptors and other cells in the retina [7]. Mutations in adherens junction proteins like crumbs 1 (CRB1) are associated with blinding diseases such as retinitis pigmentosa (RP) and Leber congenital amaurosis (LCA) [8], and ablation of proteins involved in the formation of adherens junctions like N-cadherin or catenins lead to severe retinal disorganization [8]–[11]. Although it is known that Rho GTPases are involved in the formation of adherens junctions in epithelial cells, no data are available that describe the role of Rho GTPases in the cytoarchitecture of the retina.

Rho GTPases are a group of small molecular weight GTPases that are part of the larger Ras superfamily. Of the 25 members currently identified, Ras-related C3 botulinum substrate 1 (RAC1), cell division cycle 42 homolog (CDC42) and ras homolog gene family member A (RHOA) are the classical and most well studied proteins [12]. Rho GTPases switch from their active, guanosine triphosphate (GTP) bound conformation to their inactive, guanosine diphosphate (GDP) form [13]. In the active conformation, GTPases interact with numerous effector proteins such as p21 activated kinases (PAK) and PAR proteins [14]–[17], which initiate intracellular signaling cascades for a variety of processes ranging from cellular migration to differentiation and development [18], [19].

While the role of CDC42 in epithelial cell biology is well known, its potential functions in neuronal tissues are still under investigation. Nevertheless, CDC42 has been linked to neuronal diseases like Alzheimer and Parkinson's disease through its role in cytoskeletal organization or its connection to alpha-synuclein, respectively [20], [21]. In the retina, analyses of Rho GTPases revealed their spatio-temporal expression patterns [22], [23], and the involvement of CDC42 in growth cone regulation [24]. In addition, RAC1 and CDC42 have been associated with photoreceptor degeneration and protection [25]–[28]. Here, we report on the consequences of a conditional knockdown of CDC42 for the postnatal retina. We show that absence of CDC42 during development caused improper retinal lamination, and resulted in progressive retinal degeneration, loss of function and vascular disorganization.

## Materials and Methods

### Animals and genotyping

All procedures were performed in accordance with the regulations of the Veterinary Authority of Zurich and the statement of ‘The Association for Research in Vision and Ophthalmology’ for the use of animals in research. Knockdown of *Cdc42* in the developing retina was achieved by crossing *Cdc42* floxed mice [29] with mice expressing cre recombinase under the control of the α element of the *Pax6* promoter [30]. The *Cdc42^flox/flox^;α-Cre* (from now on named *Cdc42*-knockdown, *Cdc42-KD*) and *Cdc42^flox/flox^* (from now on named control) littermates were analyzed at different embryonic and postnatal days as indicated. To study the spatial expression pattern of Cre, *α-cre* mice were crossed with the Ai6 reporter mouse [31] (a gift from Dr. Botond Roska, FMI, Basel, Switzerland) that expresses a green fluorescent protein (Zsgreen) following Cre mediated deletion of a floxed STOP cassette. *Rlbp1-GFP* mice express GFP under control of the retinaldehyde binding protein 1 (RLBP1) promoter in Müller cells and were described before [32]. *Rlbp1-GFP* mice were interbred with *Cdc42^flox/flox^;α-cre* mice to generate *Cdc42-KD;Rlbp1-GFP* mice. All mice were kept at the animal facility of the University Hospital Zurich in a dark-light cycle (12 h : 12 h) with 60 lux of light at cage level with food and water *ad libitum*.

Mice were genotyped using genomic DNA isolated from ear biopsies and the following conventional PCR conditions: initial denaturation (95°C, 5 min); 35 cycles of denaturation (95°C, 45 s), annealing (60°C, 45 s) and elongation (72°C, 45 s); final extension (72°C, 10 min). The following primers (Microsynth, Balgach, Switzerland) were used: *Cdc42* floxed (forward: 5′-ttcttcctccaacctcctgatggg-3′, reverse 5′-tgctgtgtgtggcatttgctgc-3′), cre recombinase (forward: 5′-aggtgtagagaaggcacttagc-3′, reverse 5′-ctaatcgccatcttccagcagg-3′), *Ai6* (forward: 5′-aagggagctgcagtggagta-3′, reverse 5′-ccgaaaatctgtgggaagtc-3′) and *Rlbp1-GFP* (5′- caagtgtgagagacagcattgc-3′, reverse 5′-gttgcagatatagtaccggctg-3′). PCR products were run on a 1% agarose gel for size detection.

### RNA isolation, cDNA synthesis and semi-quantitative real-time PCR

Retinas were isolated through a slit in the cornea and immediately frozen in liquid nitrogen. Total RNA was isolated using the RNeasy isolation kit (RNeasy; Qiagen, Hilden, Germany) and residual genomic DNA was removed by an additional DNase treatment. Identical amounts of RNA were reverse transcribed using oligo(dT) and M-MLV reverse transcriptase (Promega, Madison, WI, USA). Real-time PCR with specific primer pairs (Table 1), a polymerase ready mix (LightCycler 480 SYBR Green I Master Mix; Roche Diagnostics, Indianapolis, IN, USA), and a thermocycler (LightCycler, Roche Diagnostics) were used to analyze gene expression. Signals were normalized to beta-actin (*Actb*) as well as to glyceraldehyde-3-phosphate dehydrogenase (*Gapdh*), and relative expression was calculated using the comparative threshold cycle (ΔΔCT) method.

**Table 1:** Primers and conditions for real-time PCR.

Gene	Oligonucleotide Primers		Annealing temp. (°C)	Product (bp)
	Forward 5′-3′	Reverse 5′-3′		
*Actb*	caacggctccggcatgtgc	ctcttgctctgggcctcg	62	153
*Gapdh*	agcaatgcatcctgcacc	tggactgtggtcatgagccc	58	96
*Pou4f1*	cctccctgagcacaagtacc	cacgctattcatcgtgtggt	60	212
*Casp1*	ggcaggaattctggagcttcaa	gtcagtcctggaaatgtgcc	60	138
*Cdc42*	ggcggagaagctgaggacaag	agcggtcgtagtctgtcataatcctc	60	275
*Vsx2*	ccagaagacaggatacaggtg	ggctccatagagaccatact	60	111
*Edn2*	agacctcctccgaaagctg	ctggctgtagctggcaaag	60	64
*Fgf2*	tgtgtctatcaagggagtgtgtgc	accaactggagtatttccgtgaccg	62	158
*Gfap*	ccaccaaactggctgatgtctac	ttctctccaaatccacacgagc	62	240
*Gnat1*	gaggatgctgagaaggatgc	tgaatgttgagcgtggtcat	58	209
*Lif*	aatgccacctgtgccatacg	caacttggtcttctctgtcccg	60	216
*Ccl2*	ggctcagccagatgcagtta	ctgctgctggtgatcctctt	60	108
*Csf1*	gctccaggaactctccaata	tcttgatcttctccagcagc	62	119
*Rlbp1*	cctttccagtcgggacaagtatg	gggtttcctcattttccagcag	60	140
*Tnfa*	ccacgctcttctgtctactga	ggccatagaactgatgagagg	62	92

### Western blotting

Proteins from isolated retinas were extracted in 0.1 M Tris-HCL (pH 8.0) by sonication at 4°C. Protein content was determined using the Bradford assay and homogenates were mixed with sodium dodecylsulfate (SDS) buffer. Proteins were resolved by electrophoresis on 10% SDS–polyacrylamide gels and transferred to nitrocellulose membranes. Membranes were blocked in 5% nonfat dry milk (Bio-Rad, Hercules, CA, USA) in TBST (10 mM Tris/HCl [pH 8.0], 150 mM NaCl, and 0.05% Tween-20) for 1 hour at room temperature. Membranes were incubated overnight at 4°C in 5% milk (in TBST) containing a respective primary antibody (Table 2). Immunolabeled proteins were detected using HRP-conjugated secondary antibodies and visualized using the Renaissance Western Blot Detection Kit (PerkinElmer Life Sciences, Boston, MA, USA).

**Table 2:** Antibodies.

Antigen	Host	Dilution	Catalog number	Source
ACTB	Mouse	1∶1000	#5441	Sigma, St. Louis, MO, USA
AKT	Rabbit	1∶2500	#9272	Cell signaling technology, Beverly, MA, USA
p-AKT	Rabbit	1∶1000	#9271	Cell signaling technology, Beverly, MA, USA
POU4F1	Mouse	1∶100	MAB1585	Chemicon, Billerica, MA, USA
CATB	Mouse	1∶1000	610153	BD transduction laboratories, Lexington, KY
CRALBP	Rabbit	1∶500	-	John C. Saari, Univ. of Washington, USA
ERK1/2	Rabbit	1∶1000	9102	Cell signaling technology, Beverly, MA, USA
p-ERK1/2	Rabbit	1∶1000	9101	Cell signaling technology, Beverly, MA, USA
GFAP	Mouse	1∶500	G3893	Sigma, St. Louis, MO, USA
GNAT1	Rabbit	1∶1000	Sc-389	Santa Cruz Biotechnology, USA
GLUL	Mouse	1∶500	MAB302	Millipore, Billerica, MA, USA
IBA1	Rabbit	1∶500	019-19741	Wako, Neuss, Germany
JAK2	Rabbit	1∶500	#44-406	Invitrogen corporation, Camarillo, USA
p-JAK2	Rabbit	1∶250	#44-426	Invitrogen corporation, Camarillo, USA
PRKCA	Rabbit	1∶1000	P4334	Sigma Aldrich, Missouri, USA
STAT3	Rabbit	1∶1000	#9132	Cell signaling technology, Beverly, MA, USA
p-STAT3	Rabbit	1∶500	#9131	Cell signaling technology, Beverly, MA, USA

### Light and transmission electron microscopy

Eyes of postnatal mice were enucleated and fixed overnight at 4°C in 2.5% glutaraldehyde in 0.1 M cacodylate buffer (pH 7.3). Cornea and lens were removed and eyecups cut dorso-ventrally through the optic nerve head. Trimmed tissue was washed in cacodylate buffer, incubated in osmium tetroxide for 1 hour, dehydrated in a series of increasing ethanol concentrations, and embedded in Epon 812. For light microscopy (Axioplan, Zeiss, Feldbach, Switzerland) semi-thin cross sections (500 nm) were cut and counterstained with toluidine blue. For transmission electron microscopy (TEM), ultrathin sections (50 nm) were stained with uranyl acetate and lead citrate and analyzed using a Philips CM100 transmission electron microscope. Heads of embryos were isolated at E14.5 and E17.5 and fixed in 2.5% glutaraldehyde in 0.1 M cacodylate buffer (pH 7.3) for at least 24 h. Further preparation and sectioning was as described above for postnatal eyes.

### Immunofluorescence

Eyes were enucleated and fixed in 4% (wt/vol) paraformaldehyde (PFA) in 0.1 M phosphate buffered saline (PBS; pH 7.4) overnight. Cornea and lens were removed and eyecups were post-fixed in 4% PFA for an additional 2 hours before being immersed in 30% sucrose in PBS at 4°C overnight. Eyes were embedded in tissue freezing medium (Leica Microsystems Nussloch GmbH, Nussloch, Germany) and slowly frozen in a liquid nitrogen cooled 2-methylbutane bath. Retinal cryosections (12 µm) were cut dorso-ventrally, placed on slides and immersed in blocking solution (3% horse serum, 0.3% Triton X-100, 0.1 M PBS) for 1 hour at room temperature. Sections were incubated overnight at 4°C in a humidity chamber with respective primary antibodies diluted in blocking solution (Table 2). After three washing steps with PBS, the secondary antibody (coupled to Cy2, Cy3 or Cy5; Jackson immunoresearch, Suffolk, UK) was added and incubation continued at room temperature for 1 hour. After washing, cell nuclei were stained with DAPI and slides were mounted with anti-fade medium (10% Mowiol 4–88; vol/vol; Calbiochem, San Diego, CA, USA), in 100 mM Tris (pH 8.5), 25% glycerol (wt/vol), 0.1% 1,4-diazabicyclo [2.2.2] octane (DABCO)). Immunofluorescently labeled proteins were visualized using a fluorescent microscope (Axioplan, Zeiss, Switzerland).

### Retinal whole mounts

Eyes were enucleated and incubated in 2% PFA in PBS for 5 minutes. Thereafter, cornea and lens were removed and the retina carefully separated from the eyecup. The retina was cut into a “clover-leaf” shape and post-fixed in 4% PFA in PBS for 10 minutes. Whole mounted retinas were washed briefly with PBS and placed in blocking solution (3% horse serum, 0.3% Triton X-100 in PBS) for 1 hour. Retinas were incubated overnight at 4°C with either *G. simplicifolia* isolectin IB_4_-Alexa594 (Invitrogen, Basel, Switzerland) or anti-mouse glial fibrillary acidic protein (GFAP) (see Table 2 for antibody details) in blocking solution. Retinal whole mounts were washed with PBS and either directly mounted using Mowiol or further incubated for 1 hour with a secondary antibody conjugated to fluorescent dye in blocking solution. Immunolabeled proteins were visualized using a digitalized light microscope (Axiovision, Zeiss, Switzerland) or a confocal microscope (Leica SP5 Confocal Microscope). The Imaris software (Bitplane AG, Zurich, Switzerland) was used to analyze z-stacks and to generate xz-projections.

### Electroretinogram (ERG) recordings

Electroretinograms were recorded from both eyes simultaneously following published protocols [33], [34]. Briefly, mice were dark-adapted overnight and anesthetized the next day with ketamine (66.7 mg/kg) and xylazine (11.7 mg/kg). Pupils were dilated with 1% Cyclogyl (Alcon, Cham, Switzerland) and 5% phenylephrine (Ciba vision, Niederwangen, Switzerland) 30 minutes before performing single flash ERG recordings under dark-adapted (scotopic) and light adapted (photopic) conditions. Light adaptation was accomplished with low background illumination starting 5 minutes before photopic recording. Single white-flash stimulus intensities ranged from −3.7 to 1.9 log cd*s/m^2^ under scotopic and from −0.6 to 2.9 log cd*s/m^2^ under photopic conditions, divided into 12 and 8 steps, respectively. Ten responses per flash intensity were averaged with an interstimulus interval of either 4.95 seconds or 16.95 seconds (for 1.4, 1.9, 2.4, and 2.9 log cd*s/m^2^).

### Statistical analysis

Statistical analysis was performed using Prism4 software (GraphPad Inc., San Diego, USA. All data are presented as mean values ± standard deviations (SD). The number of samples (*N*) used for individual experiments is given in figure legends. Unless stated otherwise, statistical differences of means were calculated using two-way ANOVA followed by a Bonferroni post hoc test. Differences with *P*-values below 0.05 were considered significant.

## Results

### Retinal disorganization in the absence of CDC42

**Figure 1:**
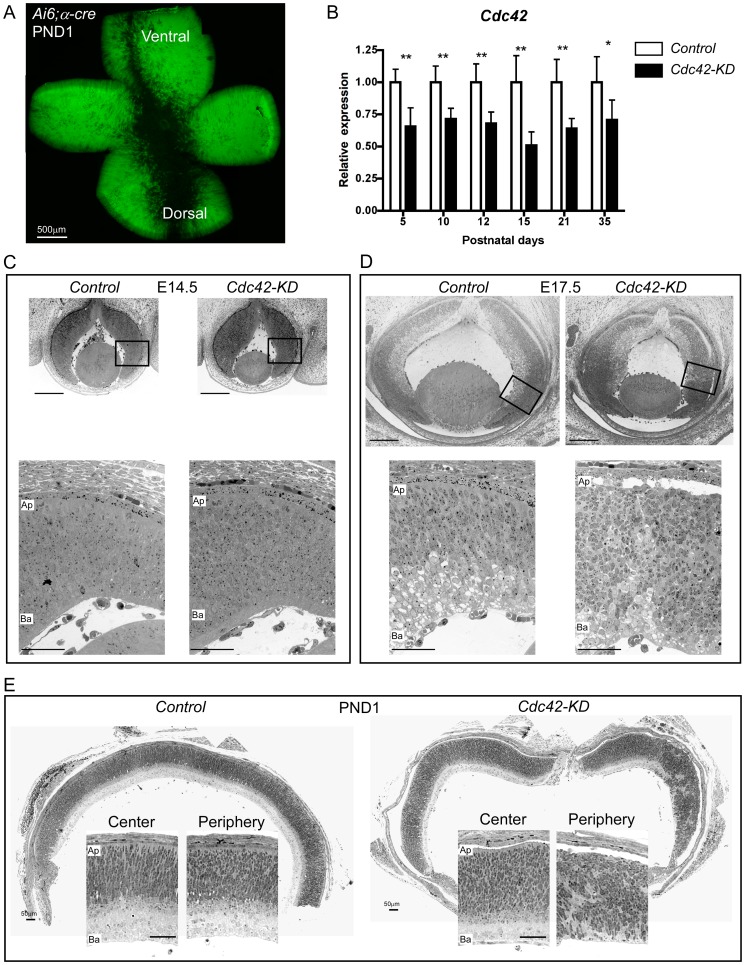
Early retinal disorganization in *Cdc42-KD* mice. (A) Retinal whole mount of a *Ai6;α-cre reporter* mouse at postnatal day (PND) 1. Cells with Cre activity express a bright green fluorescent protein (Zsgreen1). Scale bar: 500 µm. (B) Relative *Cdc42* mRNA levels in retinas of control and *Cdc42-KD* mice at indicated postnatal days. Values were normalized to *β-actin* and expressed relatively to control at each time point tested. Shown are mean values ± SD of *N* = 5 retinas. Statistical significance was calculated using one tailed t tests for each individual time point. *p<0.05, **p<0.01. (C;D) Retinal morphology of control and *Cdc42-KD* eyes at E14.5 (C) and 17.5 (D), respectively. Top panels: overview, scale bars: 200 µm. Bottom panels: magnifications of boxed areas in top panels, scale bars: 50 µm. (E) Morphology of representative retinal panoramas and sections of central and peripheral retinal regions of control and *Cdc42-KD* mice at PND1. *N* = 3 per genotype. Ap: apical, Ba: basal. Scale bars: 50 µm.

To delete CDC42 specifically in the retina we crossed *Cdc42^flox/flox^* (control) mice [29] with α-Cre deleter mice [30] and selected for *Cdc42^flox/flox^;α-Cre* (*Cdc42-KD*). α-Cre mice start to express Cre recombinase at E10.5 in retinal progenitors leading to the deletion of floxed sequences in progenitor-derived cells that localize to the periphery of the postnatal retina [30]. Cells of the retinal pigment epithelium were not affected (not shown and as reported before [30], [35]. This spatial distribution of Cre-activity was confirmed by retinal whole mounts from *Ai6;α-cre* reporter mice. Large peripheral areas were positive for Zsgreen1 already at PND1. The central retina as well as a dorso-ventral “streak”, however, were negative for fluorescence and thus lacked active Cre protein. The Cre-negative area in the ventral periphery was much narrower than the corresponding dorsal area (Fig. 1A).

Cre-activity resulted in reduced expression levels of *Cdc42* mRNA in retinas of young and adult *Cdc42-KD* mice. Analysis by real-time PCR showed that *Cdc42* levels were reduced by 35% already at PND5, the earliest time point analyzed. This reduction remained roughly constant at all ages tested (Fig. 1B). Morphological analysis of control and *Cdc42-KD* retinas showed minimal differences, if any, at E14.5 (Fig. 1C). By E17.5, however, the retinal neuroepithelium of *Cdc42-KD* mice was severely disorganized, showing disruptions in lamination (Fig. 1D). Consistent with region of Cre activity, the phenotype was most pronounced in the peripheral retina. At PND1, the phenotype in the ventral, peripheral retina persisted and revealed severe disorganization whereas the dorsal retina, which corresponds to the region with the least Cre activity, was comparable to controls (Fig. 1E).

### Loss of apically localized adherens junctions

**Figure 2:**
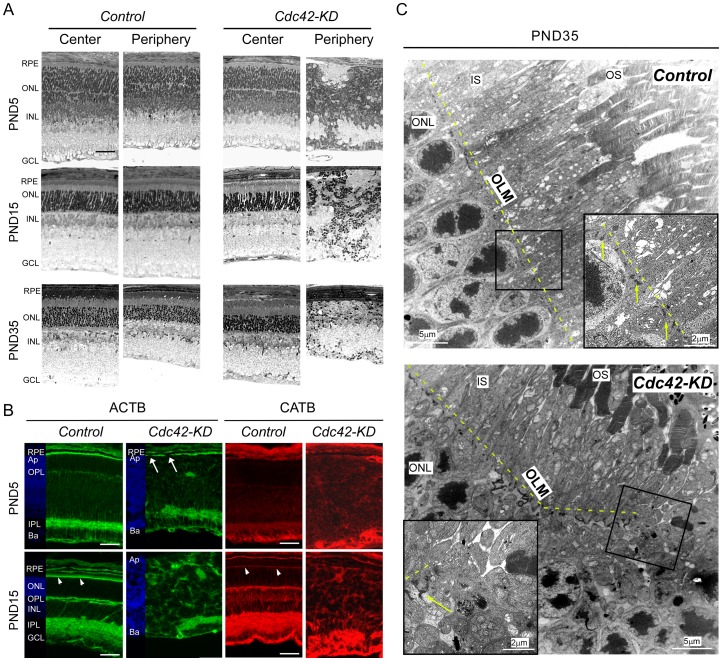
Loss of the outer limiting membrane (OLM) in the retinal periphery of *Cdc42-KD* mice. (A) Retinal morphology of central and peripheral regions of control and *Cdc42-KD* mice at indicated postnatal days (PND). (B) Immunolabeling of β-Actin (ACTB) and β-Catenin (CATB) in peripheral regions of control and *Cdc42-KD* retinas at PND5 and 15. Arrowheads point to the staining in the region of the OLM. Arrows indicate discontinuous ACTB labeling in the region of the OLM in *Cdc42-KD* retinas. DAPI image insets are used for orientation. (C) Transmission electron microscopy pictures of adherens junctions at the OLM in the retinal periphery of control and *Cdc42-KD* retinas at PND35. Insets show magnifications of boxed areas and dashed yellow lines and yellow arrows indicate the location of the OLM. Shown are representative samples of *N* = 3 for each panel. RPE: retinal pigment epithelium, ONL: outer nuclear layer, INL: inner nuclear layer, GCL: ganglion cell layer, OS: photoreceptor outer segments, IS: photoreceptor inner segments, OPL: outer plexiform layer, IPL: inner plexiform layer, Ap: apical, Ba: basal. Scale bars: 50 µm or as indicated.

The disorganization of the young retina in *Cdc42-KD* mice (Fig. 1) resulted in an almost complete absence of lamination of the older peripheral retina at PND5, 15 and 35 (Fig. 2A). Central retinal regions of *Cdc42-KD* mice, however, had preserved a tissue layering that was comparable to control retinas (Fig. 2A). Loss of tissue lamination was also observed in the absence of *β-catenin* (CATB) in mouse or of *N-cadherin* in zebrafish [9], [10]. Both proteins are part of adherens junctions at the outer limiting membrane (OLM) [36], [37], which is located at the apical side of the ONL and connects photoreceptors with processes of Müller glia cells. Since CDC42 is involved in initiation of adherens junction formation in epithelial cells [6], we investigated tissue distribution of CATB and β-Actin (ACTB) in retinas of *Cdc42-KD* mice. In control mice ACTB localized mainly to a narrow band in the apical retina, to the forming inner plexiform layer (IPL) and to the basal side of to the forming GCL at PND5 (Fig. 2B). A faint staining was also observed in the region of the forming outer plexiform layer (OPL). This staining pattern was maintained at PND15 with a more intense labeling of the OPL and in the region of the OLM and photoreceptor segments (arrowheads). In 5-day old *Cdc42-KD* mice, however, staining of the narrow band in the apical region of the peripheral retina was discontinuous (Fig. 2B: arrows), and the signal in the IPL was more diffuse and irregular. At PND15, the irregular pattern of ACTB staining was even more pronounced with the presence of ACTB-positive structures throughout the retina (Fig. 2B). Similar results were obtained for CATB. While CATB localized to different layers in control retinas with a distinct labeling in the region of the OLM at PND15 (arrowheads), staining was much more diffuse in the absence of CDC42. Notably, a distinct and continuous staining in the region of the OLM was missing in *Cdc42-KD* at PND15 (Fig. 2B). Central retinal regions of *Cdc42-KD* mice were not affected and showed ACTB and CATB stainings similar to controls (not shown).

Such unstructured signals for CATB and ACTB in *Cdc42-KD* retinas support the morphological findings that show severe disorganization of the retinal tissue (Fig. 2A). In addition, disruption or lack of staining in the region of the OLM in the apical retina suggested that the OLM and adherens junctions might be affected. Using transmission electron microscopy, adherens junctions were recognized as electron dense structures located apically to the ONL in control retinas (Fig. 2C, top panel). In *Cdc42-KD* retinas, however, adherens junctions were found only in the more central retina (Fig. 2C, left part of bottom panel). In the periphery and thus in the retinal region with Cre activity, retinal organization was lost, adherens junction structures disappeared and the OLM was discontinuous (Fig. 2C, right part of bottom panel). The disruption of the OLM can be recognized especially in the ‘transition zone’ between the central (unaffected) and the affected peripheral retina (Fig. 2C, bottom panel).

Overall, this suggests that CDC42 is vital for correct lamination of the retina and that this GTPase may be directly or indirectly required for in the formation of adherens junctions between photoreceptors and Müller glia cells. Absence of these junctions may weaken cell-cell contacts and thus contribute to the severe disorganization of the retina.

### Lack of CDC42 causes mislocalization and activation of Müller glia cells

**Figure 3:**
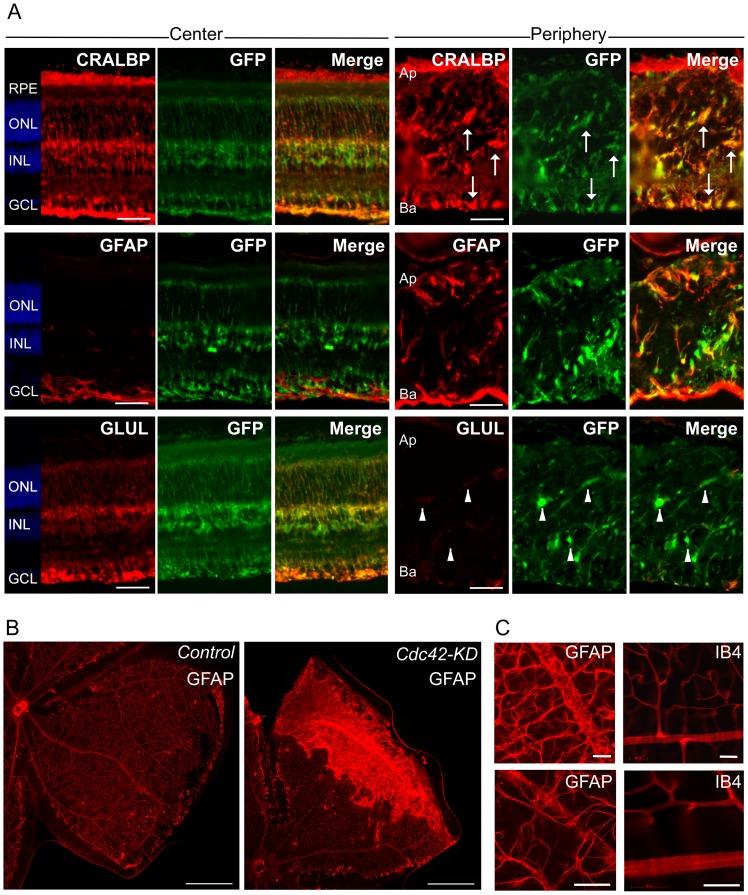
Mislocalization, misalignment and activation of Müller glia cells in Cdc42-KD retinas. (A) Retinal cryosections of central and peripheral regions of 35 days old *Cdc42-KD;Rlbp1-GFP* mice stained for cellular retinaldehyde binding protein (CRALBP), glial fibrillary acidic protein (GFAP) and glutamine synthetase (GLUL) to label Müller glia cells. Arrows indicate colocalization of CRALBP with green fluorescent protein (GFP). Arrowheads indicate minimal colocalization of GLUL with GFP. Scale bar: 50 µm. (B) Retinal whole mounts of 2-month-old control and *Cdc42-KD* mice stained for GFAP. (C) High magnifications of GFAP and isolectin IB4 stainings in whole mounts of retinas from control mice. Scale bars: 50 µm. Shown are representative samples of *N* = 3 for each panel. RPE: retinal pigment epithelium, ONL: outer nuclear layer, INL: inner nuclear layer, GCL: ganglion cell layer, GFP: green fluorescent protein, Ap: apical, Ba: Basal. Scale bar: 500 µm.

Müller cells are radial glial cells that fulfill many functions in the retina including stabilization of the tissue. Disruption of Müller cells in the postnatal retina leads to severe retinal degeneration and retinal dysplasia [38]. Since lack of CDC42 affects radial glia morphology in the cortex and leads to abnormal positioning of cortical neurons [39], we tested whether lack of CDC42 influences Müller cells in the retina. For this purpose, we crossed *Cdc42-KD* with *Rlbp1-GFP* mice, which express green fluorescent protein (GFP) specifically in Müller glia cells [32]. Sections from the central and peripheral retina of 35 days old *Cdc42-KD;Rlbp1-GFP* mice were stained with antibodies for three glial markers (cellular retinaldehyde binding protein (CRALBP), glial fibrillary acidic protein (GFAP), and glutamine synthetase (GLUL)) (Fig. 3A). The central retina showed the expected staining pattern with localization of CRALBP in the RPE, and in Müller cells stretching from the GCL to the apical side of the ONL. GFAP was only expressed in Müller cell endfeet and in astrocytes residing in the GCL. GLUL showed a similar expression pattern as CRALBP, except that no signal was present in the RPE. In the peripheral *Cdc42-KD;Rlbp1-GFP* retina, CRALBP staining also colocalized with the transgenic GFP signal from Müller glia cells (Fig. 3A: arrows), but Müller cell bodies were not correctly localized and cellular processes were not detectable. This suggests that Müller cells were present in the retina even in the absence of CDC42 but that they were mislocalized and not radially aligned in the tissue. In contrast to the central retina, GFAP was upregulated in the periphery and also colocalized with transgenic GFP protein in Müller cells (Fig. 3A). Glutamine synthetase, however, was downregulated in the peripheral retina and almost no GLUL protein was detectable in cells expressing GFP (Fig. 3A: arrowheads). Since downregulation of *Glul* expression is known to occur in activated Müller cells [40], this suggests that the peripheral retina of *Cdc42-KD* mice may contain strongly stimulated Müller glia. This is further supported by the GFAP staining pattern in retinal whole mounts of 2-month-old mice (Fig. 3B). In control retinas GFAP was mainly localized in astrocytes, which delineate vessels in the primary plexus (Fig. 3A and B). Thus, GFAP staining in whole-mounted wild type retinas results in a pattern resembling the retinal vasculature of the primary plexus as suggested by others [41]. The specificity of the GFAP staining in Fig. 1B is confirmed by higher magnifications which show that GFAP antibodies recognize radial processes of astrocytes that contact vessels (Fig. 3C). In contrast, isolectin IB4 staining only labels vascular endothelial cells resulting in a immunolabeling pattern different from GFAP-stained whole mounts when viewed at high magnification (Fig. 3C). In *Cdc42-KD* retinas, however, GFAP expression was upregulated in Müller cells of the retinal periphery (Fig. 3A) corresponding to a staining pattern in whole mounts, which may point to the formation of a gliotic scar (Fig. 3B).

**Figure 4:**
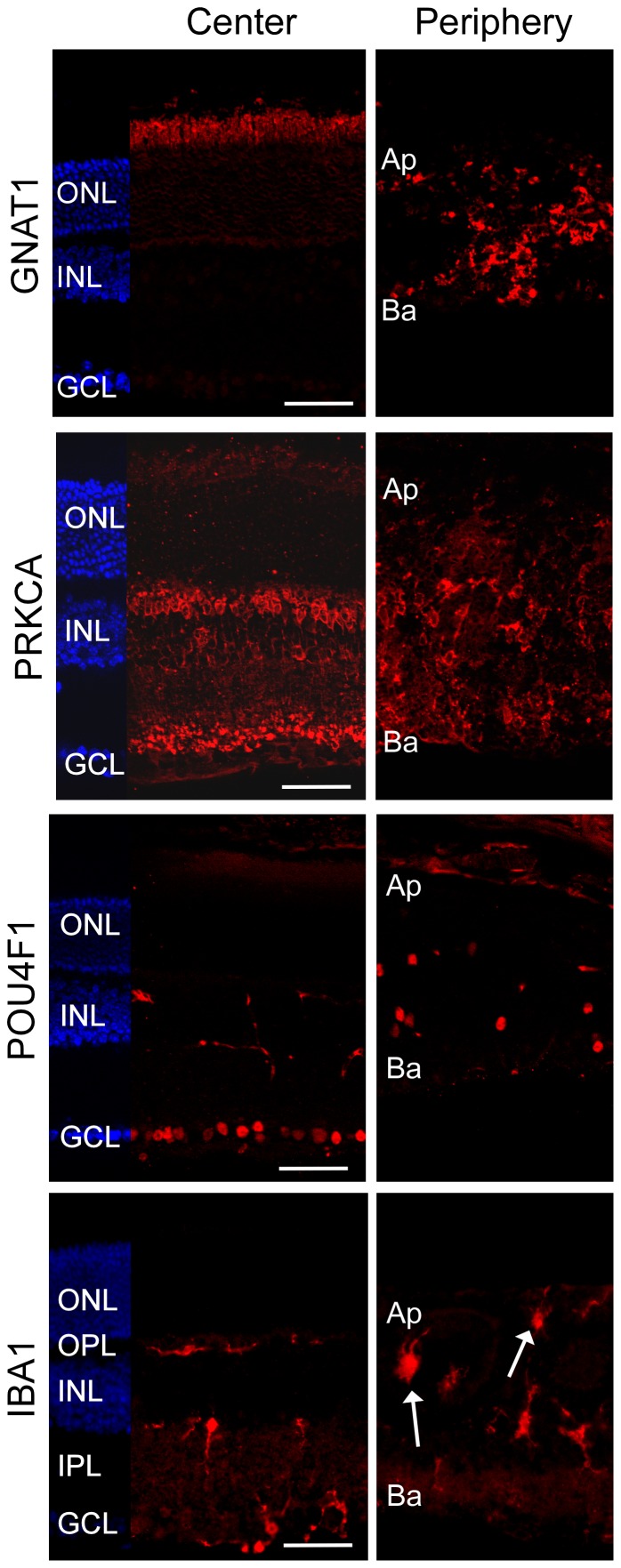
Detection of neuronal and microglial cells in adult *Cdc42-KD* retinas. Retinal cryosections of central and peripheral regions of *Cdc42-KD* mice were stained for rod photoreceptors (GNAT1), rod bipolar cells (PRKCA), ganglion cells (POU4F1), and microglia (IBA1). Shown are representative images of *N* = 3. Arrows point to amoeboid shape microglia in the periphery. ONL: outer nuclear layer, OPL: outer plexiform layer, INL: inner nuclear layer, IPL: inner plexiform layer, GCL: ganglion cell layer, Ap: apical, Ba: basal. Scale bars: 50 µm.

Immunofluorescent labeling using specific markers for rods (rod transducin, GNAT1), rod bipolar cells (protein kinase C α, PRKCA), ganglion cells (POU domain class 4 transcription factor 1, POU4F1), and microglia (ionized calcium binding adaptor molecule 1, IBA1) showed the presence of these proteins in both the central and peripheral retina of *Cdc42-KD* mice (Fig. 4). The GNAT1 signal corresponded to the location of photoreceptor outer segments, the PRKCA signal to the position of synaptic endings of rod bipolar cells and to bipolar cell bodies, the POU4F1 signal to the GCL and the IBA1 signal mainly to the plexiform layers in central retinas. In the periphery, however, signals showed an irregular pattern suggesting a severe disorganization of retinal cells expressing these markers (Fig. 4). Also, some microglia seemed to have adopted an amoeboid-like shape (Fig. 4, arrows), which is indicative of an activated state of these cells [42]. Activation of resident microglia is a common phenomenon in degenerating retinas of mice [43].

### Disorganization of the retinal vasculature in the absence of CDC42

**Figure 5:**
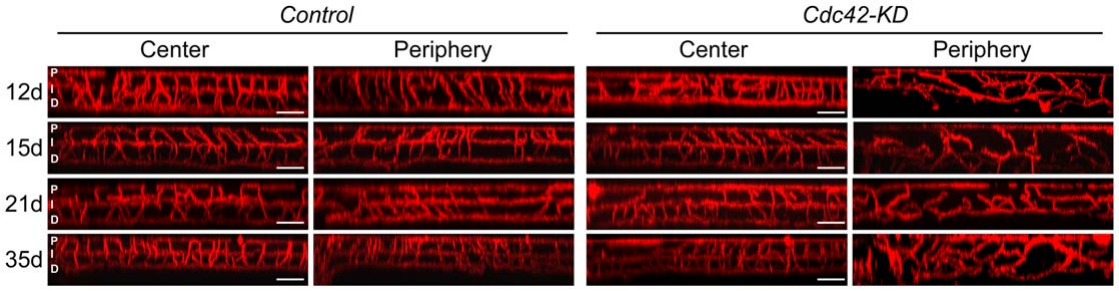
Disorganized retinal vasculature in the retinal periphery of *Cdc42-KD* mice. XZ-projection images of Isolectin IB4 labeled retinal whole mounts from control and *Cdc42-KD* mice at indicated postnatal days (d). Shown are representative samples of *N* = 3 for each genotype and time point. Scale bar: 100 µm. P: primary plexus, I: intermediate plexus, D: deep plexus.

Glia cells are important for the development of the retinal vasculature. Astrocytes provide a cellular network for growth of the primary vascular plexus, and Müller glia processes may act as guides for endothelial cell sprouting in the process of formation of the deeper vascular plexi [1], [44]. Since Müller cells were not correctly localized and processes were not aligned in a radial fashion, we expected retinal vasculature aberrations in the periphery of *Cdc42-KD* mice. To test this, retinal whole mounts were stained for isolectin IB_4_ at PND12, 15, 21 and 35, and xz-stack projections of central and peripheral retinal regions of *Cdc42-KD* and control mice were compiled (Fig. 5). Whereas all vascular plexi in central retinal regions were comparable in both mouse strains, the intermediate and deep plexi of the peripheral vasculature in retinas of *Cdc42-KD* mice were severely disturbed. Although the primary plexus apparently has formed, no discernable intermediary and deeper plexi were evident and vessels were disorganized at all ages tested. Note that in 12 day old control mice, the deep plexus had already formed but the intermediate plexus has not yet reached the far periphery, as expected (Fig. 5).

### Severe loss of retinal cells and function in the absence of CDC42

**Figure 6:**
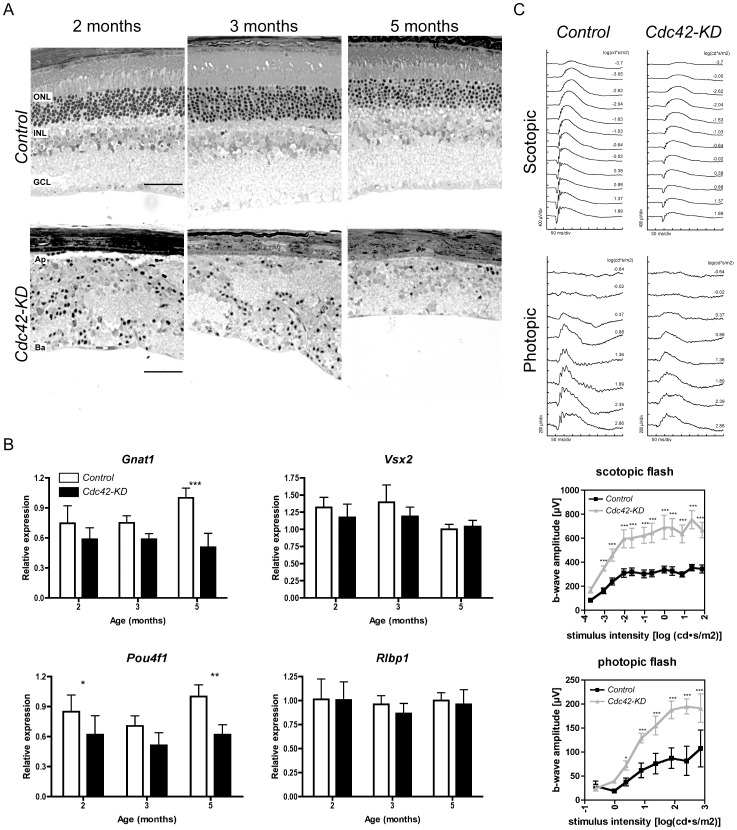
Progressive retinal degeneration and reduced retinal function in *Cdc42-KD* mice. (A) Morphology of the peripheral retina from control and Cdc42-KD mice at indicated ages. Shown are representative samples of *N* = 3. (B) Gene expression of retinal markers in retinas of control (white bars) and *Cdc42-KD* (black bars) mice at indicated ages. Expression was normalized to *Actb* and expressed relatively to control values at 5 months of age, which were set to “1”. Shown are mean values ± SD of *N* = 5 retinas. Statistical significance was calculated using two-way ANOVA with a Bonferroni post hoc test. *p<0.05, **p<0.01, ***p<0.001. (C) Scotopic (dark adapted) and photopic (light adapted) electroretinogram recordings from control and *Cdc42-KD* mice at 2 months of age. Representative original traces from individual mice are presented (top panels). Scotopic and photopic b-wave amplitudes of control and *Cdc42-KD* mice are calculated as a function of light intensity (bottom panels). *N* = 3 for each genotype. Two-way ANOVA with Bonferroni post hoc tests was used to test statistical significance. *p<0.05, ***p<0.001. ONL: outer nuclear layer, INL: inner nuclear layer, GCL: ganglion cell layer, Ap: Apical, Ba: Basal. Scale bar 50 µm.

To analyze long-term consequences of the severe retinal disorganization observed in young *Cdc42-KD* mice, we analyzed retinal morphology and expression of retinal marker genes in 2, 3 and 5-month-old mice. Whereas the RPE appeared unaffected, progressive and severe reduction of retinal thickness was observed in the periphery of *Cdc42-KD* mice (Fig. 6A). This suggested substantial loss of cells in *Cdc42-KD* mice but not in controls. To identify the cell classes that degenerated in *Cdc42-KD* retinas we tested RNA expression levels of markers for specific cell populations. Levels of *Gnat1* and *Pou4f1* were significantly reduced whereas levels of *Vsx2* and *Rlbp1* were comparable to controls (Fig. 6B). This suggests that lack of CDC42 during retinal development primarily affected survival of photoreceptor and ganglion cells, but not of bipolar and Müller glia cells, respectively.

The consequences of cell loss for overall retinal function was tested using full field electroretinography (ERG). Recordings of single flash light intensity series in 2-month-old control and *Cdc42-KD* mice showed a significant reduction of scotopic and photopic b-wave amplitudes (around 40% at highest flash intensities) in mice lacking CDC42 in the retinal periphery (Fig. 6C). This reduction in function suggests that both the rod and the cone system were affected in *Cdc42-KD* mice.

Together, these results show that the peripheral retina of *Cdc42-KD* mice exhibited progressive retinal degeneration that was also reflected by a significant loss of function.

### Activation of endogenous stress response systems in the absence of CDC42

**Figure 7:**
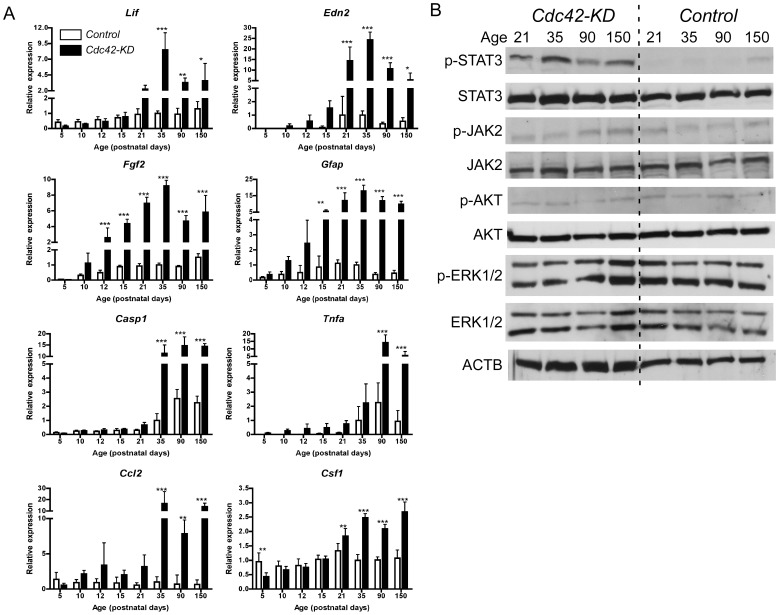
Molecular signaling in retinas of *Cdc42-KD* mice. (A) Semi-quantitative real-time PCR analysis of retinal RNA levels of control (white bars) and *Cdc42-KD* (black bars) mice at postnatal days (PND) as indicated. Gene expression was normalized to *Actb* and expressed relatively to control levels at PND35, which were set to “1”. Mean values ± SD of *N* = 5 mice are shown. If no bar is visible: expression below detection limits. Statistical significance was determined by two-way ANOVA with Bonferroni post hoc tests. *p<0.05, **p<0.01, ***p<0.001. (B) Representative Western blots of whole retinal extracts from control and *Cdc42-KD* mice (*N* = 3) at postnatal days (age) as indicated. ACTB was used as loading control.

The severe retinal disorganization in retinas of *Cdc42-KD* mice may induce endogenous response systems as seen in degenerating retinas [45]. Such a response may include both pro- and anti-survival signaling pathways. A prominent survival pathway activated in the mouse retina, is regulated by leukemia inhibitory factor (LIF) and involves endothelin 2 (EDN2) and fibroblast growth factor 2 (FGF2) [45]–[48]. To test whether this system would also be activated in the disorganized and degenerative *Cdc42-KD* retina, we used semi-quantitative real-time PCR to analyze retinal gene expression levels in mice up to 150 days of age (Fig. 7A). All expression data were normalized to *Actb*. Although ACTB has a direct role in the organization of the cytoskeleton, very similar data were obtained after normalization to expression of *Gapdh* (data not shown).

Expression of *Lif*, *Edn2*, and *Fgf2* was strongly induced in *Cdc42-KD* mice with a peak around PND35 (Fig. 7A). It is interesting to note that expression of *Fgf2* increased in *Cdc42-KD* retinas before *Lif* was induced. Since activation of *Fgf2* depends on LIF in models of photoreceptor degeneration [45], [46], this may indicate that the disorganized retina, where cells in addition to photoreceptors are affected, uses other or additional mechanisms to activate *Fgf2*. It is also interesting that *Lif* expression was at basal levels until PND21 although retinal organization was severely affected already before that time point (Fig. 2A). Whether this indicates that photoreceptor cells are not recognized as ‘injured’ in the young dysmorphic *Cdc42-KD* retina needs further exploration. Similar to *Fgf2*, expression of *Gfap* was induced early (PND15) and remained significantly elevated throughout the time period analyzed. Pro-apoptotic factors (Caspase1 (*Casp1*), tumor necrosis factor α (*Tnfa*)) and pro-inflammatory chemokines (Chemokine (C-C motif) ligand 2 (*Ccl2*), macrophage colony stimulating factor 1 (*Csf1*)) were expressed at significantly higher levels in *Cdc42-KD* retinas from PND35 or PND90, respectively, onwards (Fig. 7A).

LIF signaling during retinal degeneration is thought to act through the JAK/STAT pathway including activation of Janus activated kinase 2 (JAK2), JAK3 and signal transducers and activators of transcription 3 (STAT3) [45], [48]–[50]. Here we tested activation of STAT3, JAK2, AKT and ERK1,2, the two latter being frequently implicated in degenerative and protective processes in the retina. From these proteins only STAT3 showed clear and sustained activation through phosphorylation, with a peak at PND35 (Fig. 7B). JAK2 and ERK were not or only slightly phosphorylated at the last time point analyzed. Thus, signaling in the degenerative *Cdc42-KD* retina may not involve AKT or ERK1/2 proteins, and regulation of STAT3 phosphorylation in the *Cdc42-KD* retina may involve JAK2-independent pathways.

## Discussion

Positioning of retinal cells during development and establishment of adherens junctions are important to generate a multi-layered and correctly organized adult retina [9], [10], [51]. While it is well known that CDC42 plays a pivotal role in the formation of adherens junctions in epithelial cells [6], little was known about its functions in the developing retina. Our results show that tissue-specific inactivation of *Cdc42* leads to a dysmorphic retina characterized by a strong disorganization of the retinal architecture and the absence of obvious adherens junctions between photoreceptors and Müller glia cells, an abnormal stratification of the retinal vasculature, a loss of retinal function and an activation of endogenous signaling pathways in response to progressive retinal degeneration.

### Loss of retinal lamination in the absence of CDC42

Retinal lamination is controlled by several mechanisms including cell migration during development and the formation of adherens junctions [51]. Whether the absence of CDC42 disrupts tissue lamination because it influences migration and spatial placement of cells during retinal development, or because it is required for the generation and/or maintenance of protein junctions for cell adhesion during development and in the adult, is not yet known. Nevertheless, since the phenotype of *Cdc42-KD* mice is similar to the phenotype of retinas lacking the adherens junction protein β-catenin, CDC42 may have an important role for cell adhesion as suggested for β-catenin [10]. Furthermore, since inactivation of *Cdc42* in mature rod photoreceptors does not lead to retinal disorganization and degeneration [28], CDC42 may be particularly important during development and/or in cells different from rods. Among other cells, lack of CDC42 caused Müller glia cells to be mislocalized and misaligned. It has been shown that radial glial cells are important for migration of neurons in the developing cerebral cortex [39] and that movements of progenitor cells are involved in cell fate decision in the mouse telencephalon [52]. Therefore, mislocalization of Müller cells may have impaired movement of retinal progenitors in the developing neuroepithelium, thereby contributing to the lack of retinal lamination observed in *Cdc42-KD* mice. A developmental role of CDC42 is also supported by the observation that ablation of CDC42 in progenitor cells of the developing mouse cortex results in a gradual loss of apically localized PAR complex proteins and adherens junctions, as well as in a disturbed interkinetic nuclear migration [52]. The α-Cre driven inactivation of *Cdc42* in retinal progenitor cells already during early embryonic development may have similarly complex consequences leading to the observed phenotype. Identifying the molecular mechanisms downstream of CDC42 that lead to the proper lamination of the developing and mature retina will be important future studies.

In addition to disorganization of the retinal morphology, lack of CDC42 also affects the formation of the vascular plexi in the postnatal retina. In the normal retina, Müller cell processes span almost the entire tissue radially from the inner to the outer limiting membrane. These processes stabilize retinal structure but are also guides for the developing retinal vasculature. Angiogenic sprouts are formed from vessels of the primary plexus and growing vessels penetrate the retina along Müller cell processes to initiate development of the deeper vascular plexi [1]. The mislocalization and non-radial alignment of Müller cells in *Cdc42-KD* mice may have prevented proper guidance of growing vessels and thus correct organization of the intermediate and deep vascular plexi. In contrast, vessels of the primary plexus were less affected. Growing vessels of this plexus reached the retinal periphery around PND10 and the capillary network was similar in both control and *Cdc42-KD* retinas (data not shown). This suggests that the astrocytic network, which provides the scaffold and signals for growing vessels of the primary plexus was not strongly affected in the Cdc42-KD retina, an interpretation supported by the astrocytic GFAP labeling at the basal side of the *Cdc42-KD* retina (Fig. 3A).

### Retinal degeneration and loss of function in the absence of CDC42

Lack of CDC42 resulted not only in a disorganized retina but also in severe and progressive degeneration of retinal cells, especially of photoreceptors and ganglion cells. Since ablation of CDC42 in adult rods does not induce degeneration [28], cell death might be caused by defects in other cells or by the dysmorphic tissue environment that might not be able to sufficiently support survival of delicate cells like photoreceptors and ganglion cells. It has been proposed for example that interactions between microglia and Müller cells modulate expression of factors for photoreceptor survival [53]. In the disorganized *Cdc42-KD* retina, however, cell-cell interactions are altered, which thus may reduce trophic support and allow degeneration. Furthermore, the persistent gliosis and gliotic scar formation in *Cdc42-KD* mice may also be detrimental and contribute to retinal degeneration [54] through the secretion of pro-inflammatory cytokines such as TNFα and CCL2 [55], [56]. Expression of both of these factors was highly elevated in *Cdc42-KD* retinas.

Lack of CDC42 also resulted in strongly reduced retinal function. Since both scotopic and photopic b-wave amplitudes were similarly reduced, both the rod and the cone system were affected. Although we cannot conclusively determine from our data whether CDC42 is directly needed for function or whether the reduction in wave amplitudes is a result of retinal disorganization and cell loss. However, since rod photoreceptors do not need intracellular CDC42 for normal function [28], we hypothesize that the reduced ERG in *Cdc42-KD* is likely the consequence of the dysmorphic and degenerative retina.

### Molecular signaling in Cdc42-KD retinas

Lack of CDC42 caused cell death in the retinal periphery. It has been shown that the retina possesses endogenous mechanisms, which provide support for neuronal cells in unfavorable situations. One of these mechanisms is controlled by LIF and includes EDN2, FGF2 and members of the JAK/STAT family of proteins [45]. Members of this pathway were also induced in *Cdc42-KD* retinas. However, whereas expression of *Fgf2* may depend on LIF in models of photoreceptor degeneration [45], [46], and may be influenced by photoreceptor-derived EDN2 [47], *Fgf2* and *Edn2* expression in *Cdc42-KD* was already upregulated before *Lif* was induced. This suggests that the highly dysmorphic retina of the *Cdc42-KD* mouse activates additional signaling pathways that may culminate in the expression of survival factors independently of LIF. Such pathways may rely on STAT3, which has been shown to be neuroprotective in other systems [48], [50], but may not include AKT and ERK signaling (Fig. 7).

Interestingly, upregulation of *Tnfa*, *Casp1*, *Ccl2* and *Csf1*, genes implicated in pro-inflammatory pathways and neurodegeneration [57]–[59], followed the upregulation of protective genes with a delay. This may suggest that survival mechanisms attempted, but were not capable to provide prolonged protection for neuronal cells and to prevent retinal degeneration in *Cdc42-KD* mice.

## Conclusions

We have shown that CDC42 ablated retinas are not properly laminated, lack obvious adherens junctions between photoreceptors and Müller glia cells, and undergo severe retinal degeneration. As a consequence, retinal function is reduced, and pro- as well as anti-survival signaling pathways are activated. Since morphological alterations were obvious as early as at E17.5, CDC42 may be important already early during retinal development for establishing a proper retinal architecture. CDC42 may possibly influence migration of cells to their correct spatial location in the developing retina, and may be required for the generation of adherens junctions between photoreceptors and Müller cells in the OLM to stabilize the retinal tissue. Investigating the CDC42 interaction partners involved in these processes may not only contribute to our understanding of developmental processes in the retina but may also help to unravel mechanisms of retinal pathologies affecting tissue integrity.
